# Factors Associated with Patient Satisfaction of Community Mental Health Services: A Multilevel Approach

**DOI:** 10.1007/s10597-019-00449-x

**Published:** 2019-09-14

**Authors:** Niccolò Stamboglis, Rowena Jacobs

**Affiliations:** 1 The Pool, City, University of London, St John Street, London, EC1V 4PB UK; 2 Calle del Magazen 5589, 30120 Venice, Italy; 3 Centre for Health Economics, University of York, Heslington, York, YO10 5DD UK

**Keywords:** Community mental-health services, Patient satisfaction, Multi-level modelling, Ordered probit model

## Abstract

Community care is increasingly the mainstay of mental healthcare provision in many countries and patient satisfaction is an important barometer of quality of patient care. This paper explores the key factors associated with patient satisfaction with community mental health services in England and then compares providers’ performance on patient satisfaction. Our analysis is based on patient-level responses from the community mental health survey, which is run annually by the Care Quality Commission (CQC) for the years 2010 to 2013. We perform a repeated cross-section analysis, identifying factors associated with patient satisfaction via a multi-level ordered probit model, including both patient- and provider-level variables. We identify hospital-specific effects via empirical Bayes estimation. Our analysis identifies a number of novel results. First, patient characteristics such as older age, being employed, and being able to work, are associated with higher satisfaction, while being female is associated with lower satisfaction. Service contact length, time since last visit, condition severity and admission to a mental health institution, are all associated with lower satisfaction. Second, treatment type affects satisfaction, with patients receiving talking therapies or being prescribed medications being more satisfied. Third, care continuity and involvement, as proxied by having a care plan, is associated with higher satisfaction. Fourth, seeing a health professional closer to the community improves satisfaction, with patients seeing a community-psychiatric nurse, a social worker or a mental-health support worker being more satisfied. Finally, our study identifies the need for service integration, with patients experiencing financial, accommodation, or physical health needs being less satisfied. At a provider level, we find a negative association between the percentage of occupied beds and satisfaction. We further identify significant provider-specific effects after accounting for observable differences in patient and provider characteristics which suggests significant differences in provider quality of care.

## Introduction

Internationally the provision of mental health services saw a paradigm shift away from institutional models of care towards care being provided in the community (Heller 1989; World Health Organization 1990). Allowing patients to be closer to their communities aligns with the objective of focusing on empowerment, involvement and recovery (Fitzsimons 2002; Tait and Lester 2005). Additionally, care in the community can help foster more integrated care (Frank and Kamlet 1989; Laugharne and Priebe 2006), reduced hospital time and an increased focus on patients’ needs (William 1993).

Traditionally, community mental health services include aspects of both mental healthcare—such as treatment, crisis care and preventative care, and social care—such as day-to-day support around managing work, relationships, personal care, and housing—or any combination of the two (Burns 2004). Depending on the healthcare system, access to services generally requires the assessment of the care needs by an appropriate professional (Mind 2013). The attendance of those needs might include a variety of care professionals with care being performed in single episodes or via longer-term service contact in the community.

Patient satisfaction with services is generally considered a key component of quality of care (Cleary and McNeil 1988; Edlund et al. 2003). Patient satisfaction affects clinical outcomes, patient retention, and medical malpractice claims. It also affects the timely, efficient, and patient-centered delivery of care (Prakash 2010). It is therefore a vital measure for health services to monitor and is often included as an important indicator of quality of mental health services (Ruggeri et al. 2007). Variation in service delivery, along with differences in patients’ needs, implies that patient satisfaction in community mental health might vary considerably across individuals and providers (Raleigh et al. 2007; Ruggeri et al. 2003).

This paper explores the determinants of patient satisfaction with community mental health services in England performing a multi-level repeated cross-section analysis of individual responses to the Care Quality Commission (CQC) community mental health survey for the years 2010 to 2013. The community mental health survey provides a national sample of the views of the national population of community mental health patients on care received. Our analysis explores the effect that patient characteristics and provider-specific variables might have on patient satisfaction. The multi-level structure of our dataset allows us to explore the presence of provider-specific effects.

Our paper makes a number of novel contributions. First our work adds to the still limited literature applying multi-level techniques to the analysis of patient satisfaction. Second, by estimating provider-specific effects, our analysis expands the current knowledge of the impact of unobservable factors such as the quality of hospital management, on patient satisfaction. Third, our work expands the body of literature by adopting a longitudinal analysis (repeated cross-sections) of patient satisfaction. Fourth, our analysis allows us to study patient satisfaction with care provided in the community. Lastly, the richness of our dataset allows us to focus on aspects which are beyond traditional care provision, such as exploring the role of patients’ needs and the type of care professional in driving satisfaction.

## Literature on Determinants of Patient Satisfaction in Mental Health Services

We explored the broader literature on the key determinants of patient satisfaction for mental health care, not just specifically community services, and identified four key areas as determinants of patient satisfaction, namely: (i) patient characteristics, (ii) access to services, (iii) the relationship with the care professional, and (iv) characteristics of services provided.

A summary of the specific elements included in these categories and their identified effect on patient satisfaction is reported in Table 1. The table shows the area of care to which studies refer (column 1), the specific factor identified by individual studies (column 2), and the sign of the factor’s effect on patient satisfaction (column 3). As indicated, a variety of factors might affect patient satisfaction and these might depend on the study design.

**Table 1:** Factors affecting patient satisfaction as identified in the literature

Key area	Specific factor	Identified effect
Patient characteristics	*Gender* [*female*]	+ Bjorngaard et al. (2007), Robillos et al. (2014)
		− Desai et al. (2005)
	*Age* [*older*]	+ Bjorngaard et al. (2007, 2012), Ford et al. (2013), Raleigh et al. (2007), Robillos et al. (2014) and Rosenheck et al. (1997)
		− Eytan et al. (2004)
	Disability [none]	+ Desai et al. (2005)
	Disability/medical comorbidities	− Holcomb et al. (1998) and Kilbourne et al. (2006)
	*Ethnicity* [*White*]	+ Swanson et al. (2007)
	*Ethnicity* [*non-White*]	− Boydell et al. (2012)
	Social class [lower]	− Boydell et al. (2012)
	Relationship status [single]	− Gigantesco et al. (2002)
	Relationship status [married]	+ Desai et al. (2005)
	Social relationships/support	+ Blenkiron and Hammill (2003) and Swanson et al. (2007)
	*Employment status* [*employed*]	+ (Holcomb et al. 1998)
		− Edlund et al. (2003) and Kilbourne et al. (2006)
	Patient status: inpatient	− Gigantesco et al. (2002)
	Psychosis diagnosis	− Boydell et al. (2012), Ford et al. (2013), Gebhardt et al. (2013) and Gigantesco et al. (2002)
	Low psychiatric severity	+ Bjorngaard et al. (2007)
	*Better subjective mental health*/*initial level of functioning*	+ Bjorngaard et al. (2007), Edlund et al. (2003), Ford et al. (2013), Holcomb et al. (1998), Robillos et al. (2014), Rosenheck et al. (1997) and Smith et al. (2014)
		− Ford et al. (2013), Gigantesco et al. (2002) and Raleigh et al. (2007)
Access to services	*Service convenience*	+ Robillos et al. (2014 and Sohn et al. (2014)
	Waiting times	− Robillos et al. (2014) and Swanson et al. (2007)
	Lack of personal support to access	− Kilbourne et al. (2006)
	Involuntary admission	− Strauss et al. (2003) and Smith et al. (2014)
	Psychiatric referral	+ Eytan et al. (2004)
	*Previous hospitalization*	+ Eytan et al. (2004)
		− Kilbourne et al. (2006) and Raleigh et al. (2007)
	Readmission intensity	− Druss et al. (1999) and Raleigh et al. (2007)
	*Contact length*	+ Rosenheck et al. (1997)
		− Gigantesco et al. (2002)
	Previously refused medication	− Strauss et al. (2003)
Relationship with care professional	Positive patient/care professional transactions	+ Baronet and Gerber (1997), Brunero et al. (2009), Pickett et al. (1995) and Smith et al. (2014)
	Therapist perceived as skilful	+ Pickett et al. (1995)
	*Team attitude*	+ Bjorngaard et al. (2007)
	Be listened to/respect for patients opinions	+ Baronet and Gerber (1997) and Pellegrin et al. (2001)
	Feeling safe and secure	+ Brunero et al. (2009)
	*Involvement*	+ Jorgensen et al. (2009), Sohn et al. (2014) and Swanson et al. (2007)
	Staff availability	+ Baronet and Gerber (1997), Robillos et al. (2014) and Sohn et al. (2014)
	*General support received*	+ Gebhardt et al. (2013) and Jorgensen et al. (2009)
	Quality of life	+ Blenkiron and Hammill (2003)
	*Financial strain*	− Kilbourne et al. (2006)
	Living alone	− Raleigh et al. (2007)
Characteristics of services provided	Support on discharge	+ Brunero et al. (2009)
	Perceived treatment quality	+ Edlund et al. (2003) and Sohn et al. (2014)
	*Perceived treatment benefit*/*helpfulness*	+ Brunero et al. (2009), Ford et al. (2013) and Pellegrin et al. (2001)
	Positive treatment outcome	+ Bjorngaard et al. (2007), Gebhardt et al. (2013), Holcomb et al. (1998), Robillos et al. (2014) and Smith et al. (2014)
	Pharmacologic disturbances	− Gebhardt et al. (2013)
	Location convenience	+ Pickett et al. (1995)
	Positive ward atmosphere/milieu	+ Jorgensen et al. (2009)
	Specialised facilities: mental health	− Rosenheck et al. (1997)
	*Larger facilities*	− Rosenheck et al. (1997)

A positive (negative) sign indicates that the study identified the factor as having a positive (negative) association with patient satisfaction. Italics factors identified in the literature were included in our model (see Table 2)

We also examined the literature on methodological approaches used to identify the determinants of patient satisfaction. These saw a considerable development over time, with initial studies using correlation analysis and more recent ones using statistical techniques such as multivariate regression and factor analysis (Rosenheck et al. 1997; Sohn et al. 2014). Recent studies identified complex interactions between factors influencing patient satisfaction at both a patient and provider level using multi-level analysis (Bjorngaard et al. 2007). From a methodological perspective the vast majority of studies have used cross-sectional analysis, with only a minority of studies focusing on longitudinal data analysis (Ruggeri et al. 2004).

## Data

We use patients’ responses to the English community mental health survey for the years 2010 to 2013 (Care Quality Commission 2010a, b). The community mental health survey is a national survey run by the English hospital regulator the CQC to capture key aspects of patient experience with care, including overall satisfaction. With an average of 13,000 annual respondents and a 31.5% response rate, this survey measures the experience of a sample of the national population of community mental health service users in England (Care Quality Commission 2010a, b).^1^ We focus on the years 2010 to 2013 as the surveys were comparable.

Each year all NHS Mental Health Trusts (hereafter referred to as hospitals)^2^ which provide secondary mental health services, including community care, are requested by the CQC to take part in the survey. Each hospital is required to identify 850 eligible patients from their records. Eligibility requires patients to have received specialist care for a mental health condition and to be seen in the community during the sampling period.

Excluded patients, according to the 2010 eligibility criteria, were those seen only once for an assessment, patients receiving drug and alcohol, learning disability, or specialist forensic services, current inpatients, and patients who only see their GP for their mental health condition. Patients also needed to be at least 18 years old (16 years old prior to 2012).

Data used in this analysis were downloaded in raw format from the UK Data Archive (Care Quality Commission 2010a, b). Details of our data cleaning process are reported in the Appendix. With the exception of ethnicity variables, which are only reported at the hospital level, all data used in the analysis are unweighted by age and gender. To avoid potential bias from a high prevalence of specific population groups in a given hospital, we control for both age and gender effects in the models.

Our analysis focused on questions which remained consistent across years. Similarly, we kept hospitals that participated in the survey in all years (52 out of 59). One further hospital was removed as hospital-level variables for that organisation were missing. This left us with 51 providers across all 4 years.

### Dependent Variable and Covariates

Our dependent variable is overall satisfaction with care measured on a six-point scale from “Very Poor” to “Excellent” until 2012 and on a 10-point scale from 0 (“Very Poor”) to 10 (“Very Good”) in 2013. To ensure comparability of overall satisfaction across years, 2013 results were mapped into the previous years’ six-point scale with 1 indicating a “Very Poor” experience, and 6 indicating an “Excellent” experience.

To minimise potential bias in translating 2013 satisfaction responses on a 6 point scale, we created a 10-to-6 mapping that minimised the distance to the average satisfaction score for the years 2010 to 2012. We aimed to reproduce a 2013 satisfaction score which on average looked like the previous 3 years. Two alternative approximations of 2013 satisfaction were computed, with mapping 2 being slightly more conservative on high scores compared to mapping 1.^3^ We used the first mapping as the base case for our analysis and the second mapping for sensitivity analysis (cfr Overall Satisfaction Mapping in the Appendix). To check for potential bias introduced by this mapping, we estimated a version of the model excluding 2013 observations (not reported).

We sought to cover as many of the factors under each of the four key areas identified in the literature (Table 1) as potential covariates in the model. The included factors are in italics in Table 1. Of the patient characteristics reported in Table 1 our analysis included gender, age and employment status. Gender was coded as a dummy variable with one indicating female. Age was captured by the survey in four different bands (under 35, 36–50, 51–65, over 65). Employment variables were registered in the survey as a “tick all that apply” option. We used dummy variables for employed, student, and voluntary work. Dummies for “retired” and “unemployed” were removed as they were correlated with age and ethnicity respectively.

A dummy variable was also used to indicate a patient’s ability to work, with one indicating being able to work. Self-reported mental health was coded on a scale from 1 to 6, with 1 indicating a “Very Poor” and 6 indicating an “Excellent” mental health status.

Of the access variables, listed in Table 1, we included length of contact with services and time passed since last contact. The former was coded on a 0 to 3 scale, with 0 indicating “less than 1 year”, 1 indicating “1 to 5 years”, 2 indicating “6 to 10 years” and 3 indicating “more than 10 years”. The other contact with services variable was coded on a 0 to 4 scale with 0 indicating “in the last month”, 1 indicating “1–3 months ago”, 2 indicating “4–6 months ago”, 3 indicating “7–12 months ago” and 4 indicating “more than 12 months ago”. A dummy variable indicating admission to a hospital for a mental health condition in the last 12 months was used as an indicator of previous hospital admissions.

Of the characteristics of services provided type variables listed in Table 1, we included dummies indicating whether patients received prescribed medications or talking therapies. We considered these variables as proxies for perceived treatment benefit.

Of the relationship with care professional variables in Table 1, we included dummies indicating respondents’ having a care plan as a proxy of involvement, and the support received on physical, accommodation and financial needs as proxies for general support received and financial strain.

In addition we included dummies to indicate the type of care professional the patient last interacted with. These included community psychiatric nurse, social worker, psychiatrist, mental health support worker, occupational therapist, and an ‘other’ care professional category.

We also included a number of hospital-level characteristics. Among patient characteristics reported in Table 1, we included the hospitals’ ethnicity composition of survey respondents, allowing us to account for potential lower satisfaction experienced by minority ethnic groups (Boydell et al. 2012; Ford et al. 2013). Ethnicity data was only available as a hospital-level aggregate weighted by age and gender.

Among the service characteristics listed in Table 1, we accounted for hospital size (Rosenheck et al. 1997) by including the total number of full time equivalent staff (medical and non-medical) as obtained from the NHS workforce statistics. This variable has been aggregated to an annual level from monthly data. Logs were taken to avoid scaling issues. The percentage of utilised hospital beds was also included as a proxy for service efficiency.

We included the percentage of hospital staff members reporting experiencing work-related stress in the last 12 months to account for potential effects of work-related stress on patient satisfaction. Stress level statistics were obtained from the NHS Staff Survey (Care Quality Commission 2010b). We interpreted this variable as influencing team attitudes from Table 1.

In addition to factors identified in the literature, we accounted for other factors affecting hospitals’ care delivery by incorporating the mental health reference cost index (MHRCI). MHRCI measures the actual cost of a hospital’s casemix compared to the national average casemix. We interpret MHRCI as an efficiency measure potentially affecting care delivery. MHRCI was the only hospital-level indicator that was not time-varying.

We then included a number of dummies to indicate which hospitals have Foundation Trust status, a measure of greater autonomy given to better performing providers. We also included year and commissioning region dummies. Our analysis aimed to include population deprivation, however this measure ended-up being collinear with ethnicity variables, therefore we removed it from the analysis. To ensure consistency across estimated models, we kept observations with no missing data across the various model specifications we ran. Our final dataset had 28,288 observations.

## Methodology

### Modelling of Determinants

We used a multi-level ordered probit model to estimate the probability of a given patient being assigned a specific satisfaction score, conditional on a set of confounders. We selected the probit model as it is the standard reference econometric specification to be used when modelling binary dependent variables. This approach models the inverse standard normal distribution of the dependent variable as a function of its covariates, via an underlying latent class model.

Contrary to ordinary least squares, probit models allows one to have estimated probabilities strictly between 0 and 1 (Woolridge 2010, Chap. 17). Our analyses are based on a repeated cross-section of survey data across single years.

Our model can be written as:

1 $$Y_{ijt} = m\quad {\text{if}}\;k_{m - 1} < y_{ijt} \le k_{m} ,\quad m = 1, \ldots ,6.$$

Our threshold values are unknown and therefore they are estimated from the data (Woolridge 2010). This threshold model relates the ordinal outcome to an unobservable underlying variable indicating patients’ overall satisfaction with the care they received. We assume this underlying latent variable to be continuous. What we observe is patient-reported overall satisfaction with care which we code as an ordered variable.

The latent satisfaction with care *y*_*ijt*_ can then be described by the following equation:

2 $$y_{ijt} = \beta_{1} x^{\prime}_{1,ijt} + \beta_{2} x^{\prime}_{2,jt} + u_{j} + e_{ijt} ,$$

where $x^{\prime}_{1,ijt}$ represents patient characteristics, $x^{\prime}_{2,jt}$ represents hospital-level variables, *u*_*j*_ represents a hospital-specific random term, and *e*_*ijt*_ is a normally distributed error term with mean 0 and variance σ^2^. We use the index *i* to refer to patients, and *j* to refer to hospitals.

We checked for collinearity among our covariates by computing Pearson correlations and by running factor analysis. Collinear variables were removed from the analysis. Survey questions with a high number of missing values were also removed.

We ran three different models. Models M0–2 represent alternative multilevel ordered probit models. Model M0 is a reference empty model including only year- and region-specific dummy variables. Model M1 allows for patient-specific characteristics. Model M2 allows for patient- and hospital-specific characteristics.

We provide an interpretation of the estimated coefficients of the ordered probit model by computing the increase in probability of observing an at least “Good” evaluation of overall satisfaction following a unitary increase in our explanatory variables (Greene 2002). Our marginal effects are computed at the average value of other explanatory variables.

We estimate the multilevel categorical probit model using the *clmm* function of the R package ORDINAL (Christensen 2011). The ordinal package allows us to estimate cumulative link (mixed) models via maximum likelihood. Mixed models are fitted with the Laplace approximation and adaptive Gauss-Hermite quadrature.

### Sensitivity Analysis

We ran a number of alternative models to check for model robustness. Our alternative models included a linear model, a simplified probit model collapsing satisfaction results into two categories (an “Excellent” and “Very Good” category, versus all other responses), a multilevel ordered probit model including a varying slope in the number of full time equivalent staff to test whether hospitals are affected differently by variations in staff numbers. To check for any bias in our transformation of patient satisfaction in 2013 we estimated a multilevel ordered probit model including data for the years 2010 to 2012 only.

### Analysis of Variance and Hospital Performance Comparison

We compare the estimated effect that individual hospitals have on the unobserved underlying patient satisfaction using Empirical Bayes techniques (Skrondal and Rabe-Hesket 2009). Empirical Bayes predictions are obtained using the prior distribution of a single hospitals’ random effects combined with the likelihood of obtaining the posterior distribution of the random effects given the observed response variables. Empirical Bayes estimates allow us to order hospitals by their base effect on patient satisfaction while all other confounders have been accounted for. We compute hospitals’ random effects as posterior modes of the distribution for the random effects given the observed data and the estimated model parameters. In our analysis we plot the posterior modes together with their 95% confidence intervals, obtained by multiplying the estimated conditional variance by the z-score corresponding to a 5% confidence level of a normal distribution (1.96%). Our Empirical Bayes have been obtained using the R function *ranef*, while the conditional variance has been obtained using the function *condVar*.

## Results

We plotted the mean of patients’ overall satisfaction across all years and across commissioning regions (see Fig. 3) and averaged across all hospitals (see Fig. 4,^4^). Overall satisfaction appeared to be comparable across years and regions, with some variation evident across hospitals.

### Modelling of Determinants

Correlation analysis identified patient satisfaction being correlated with having a care plan, support received from services for specific needs, and variables associated with relationships with care professionals, such as being listened to. Variables relating to relationships with care professionals and service support were also positively correlated with one another. Positive correlation was also present between older age (over 66) and being retired, between the London dummy and the ethnicity variables, and between unemployed and ethnicity variables. Lastly, we identified a positive correlation between the staff work-related stress variable and the 2013 dummy. To avoid collinearity we removed the variables being retired, being unemployed and relational aspects of care variables from our analysis.

Factor analysis identified the following factors: service support for specific needs, ethnicity, relational aspects of care, age, employment, being admitted to hospital, region, and being seen by a health care professional. Factor analysis also identified a factor affecting relational aspects of care and overall satisfaction simultaneously.

We interpret the potential collinearity between relational aspects of care and overall satisfaction as an indication of endogeneity via the potential presence of a common unobservable factor affecting both variables simultaneously. Including endogenous covariates in the probit model might lead to spurious results (Woolridge 2010). Although relational aspects of care might be a factor associated with patient satisfaction, the presence of both significant correlation with other covariates and the presence of an unobservable common factor with the dependent variable might lead to bias in the estimated results. For these reasons we decided to remove relational aspects of care from our analysis.

Table 2 summarises the descriptive statistics for all variables in our final estimation sample with reference categories in brackets.

**Table 2:** Descriptive statistics

N = 28,288					
Category	Variable	Mean	St. Dev.	Min	Max
Dependent variable	Overall satisfaction (1 = “Very Poor”, 6 = “Excellent”)	4.61	1.33	1	6
Explanatory variables					
Year	[2010]	0.26	0.44	0	1
	2011	0.27	0.44	0	1
	2012	0.26	0.44	0	1
	2013	0.22	0.41	0	1
Region	North	0.3	0.46	0	1
	South	0.22	0.42	0	1
	Midlands and East	0.32	0.47	0	1
	[London]	0.16	0.37	0	1
Patient-level characteristics					
Gender	[Female]	0.58	0.49	0	1
	[18–35]	0.17	0.37	0	1
Age	36–50	0.29	0.37	0	1
	51–65	0.26	0.45	0	1
	> 66	0.29	0.45	0	1
Employment status	Employed	0.14	0.35	0	1
	Student	0.02	0.47	0	1
	Voluntary	0.07	0.26	0	1
Ability to work	Being able to work (0 = “No”, 1 = “Yes”)	0.61	0.24	0	1
Mental health status	(1 = “Very Poor”, 6 = “Excellent”)	3.31	1.26	1	6
Contact with services	Length of contact with services (0 = “Less than 1 year”, 3 = “More than 10 years”)	1.64	1.18	0	3
	Last contact with services (0 = “In the last month”, 4 = “More than 12 months ago”)	0.71	1.02	0	4
Admitted	(0 = “No”, 1 = “Yes”)	0.13	0.34	0	1
Therapy	Prescribed medications (0 = “No”, 1 = “Yes”)	0.9	0.09	0	1
	Talking therapies (0 = “No”, 1 = “Yes”)	0.41	0.24	0	1
Care plan	Having a care plan (0 = “No”, 1 = “Yes”)	0.74	0.19	0	1
Specific needs	Physical health need (0 = “No”, 1 = “Yes”)	0.71	0.21	0	1
	Accommodation need (0 = “No”, 1 = “Yes”)	0.27	0.2	0	1
	Financial need (0 = “No”, 1 = “Yes”)	0.52	0.25	0	1
Health professional	Community psychiatric nurse	0.33	0.22	0	1
	Social worker	0.08	0.08	0	1
	Psychiatrist	0.25	0.19	0	1
	Mental health support worker	0.14	0.12	0	1
	Occupational therapist	0.03	0.03	0	1
	[Other health professional]	0.09	0.29	0	1

Category	Variable	Source	Mean	St. Dev.	Min	Max
Hospital-level characteristics						
Ethnicity	White	CQC	0.88	0.12	0.35	1
	Mixed	CQC	0.01	0.02	0	0.07
	Asian	CQC	0.04	0.05	0	0.25
	Black	CQC	0.03	0.05	0	0.3
	[Other]	CQC	0.04	0.03	0	0.15
Capacity	FTE staff	NHS England	4.3	0.98	0	5.32
	Percentage occupied beds	NHS England	0.87	0.06	0.69	0.99
Efficiency	MHRCI	Department of Health	1.04	0.25	0.46	3.53
Staff	Staff work-related stress	NHS England	0.37	0.01	0.18	0.53
Hospital status	Foundation Trust status	Care and Quality Commission	0.72	0.45	0	1

Reference category is given in square parentheses. Employment status does not include a reference variable as these variables were in multiple response format. Health professional variables does not sum to 1 as pre-2012 answer “psychologist” was removed for consistency

Table 3 provides the results of our multilevel models.

**Table 3:** Estimation results

	Empty model (M0)		Patient characteristics model (M1)		Patient and hospital characteristics model (M2)	
N obs	28,288		28,288		28,288	
Variable	Coefficient	Standard error	Coefficient	Standard error	Coefficient	Standard error
2011	− 0.01	0.02	− 0.02	0.02	− 0.02	0.02
2012	0.02	0.02	− 0.01	0.02	− 0.02	0.03
2013	0.2***	0.02	0.24***	0.02	0.23***	0.04
North	0.15***	0.03	0.13***	0.03	0.11*	0.04
South	0.04	0.03	0.03	0.03	− 0.02	0.05
Midlands and East	0.09***	0.03	0.1**	0.03	0.07	0.04
Patient-level characteristics						
Female			− 0.03**	0.01	− 0.03*	0.01
Mental health status			0.28***	0.01	0.27***	0.01
Admitted			− 0.17***	0.02	− 0.17***	0.02
Age 36–50			0.21***	0.02	0.21***	0.02
Age 51–65			0.27***	0.02	0.27***	0.02
Age over 66			0.42***	0.02	0.42***	0.02
Employed			0.06***	0.02	0.06**	0.02
Student			0.03	0.04	0.03	0.04
Voluntary			− 0.03	0.02	− 0.03	0.02
Length of contact with services			− 0.08***	0.01	− 0.08***	0.01
Last contact with services			− 0.18***	0.01	− 0.18***	0.01
Therapy: prescribed medications			0.14***	0.02	0.14***	0.02
Therapy: talking therapies			0.31***	0.01	0.31***	0.01
Having care plan			0.43***	0.02	0.43***	0.02
Being able to work			0.09***	0.02	0.09***	0.02
Physical health need			− 0.08***	0.01	− 0.08***	0.01
Accommodation need			− 0.19***	0.02	− 0.19***	0.02
Financial need			− 0.13***	0.01	− 0.13***	0.01
Community psychiatric nurse			0.17***	0.02	0.17***	0.02
Social worker			0.06*	0.03	0.06*	0.03
Psychiatrist			0.03	0.02	0.03	0.02
Mental health support worker			0.12***	0.02	0.12***	0.02
Occupational therapist			0.05	0.04	0.05	0.04
Hospital-level characteristics						
White					− 0.20	0.44
Mixed					0.4	0.81
Asian					− 0.71	0.57
Black					− 0.18	0.56
FTE staff					− 0.01	0.01
Percentage occupied beds					− 0.30*	0.15
MHRCI					0.05	0.03
Staff work-related stress					0.18	0.21
Foundation Trust status					− 0.01	0.02

Threshold	Estimate	Standard error	Estimate	Standard error	Estimate	Standard error
Threshold coefficients						
1|2	− 1.78	0.03	− 0.67	0.05	− 1.11	0.47
2|3	− 1.32	0.03	− 0.13	0.05	− 0.57	0.47
3|4	− 0.78	0.03	0.50	0.05	0.06	0.47
4|5	− 0.20	0.03	1.16	0.05	0.72	0.47
5|6	0.62	0.03	2.08	0.05	1.65	0.47
Diagnostics						
LogLik	− 42,976.77		− 40,079.39		− 40,071.62	
AIC	85,977.55		80,228.79		80,231.25	

Ordered probit models. 2010 is the reference year, male is the reference gender, age < 35 is the reference age, “other ethnicity” is the reference ethnicity status. M0 represents the empty model. M1 allows for patient characteristics. M2 allows for both patient- and hospital-level characteristics

Significance is *p < .05. **p < .01, ***p < .001

By computing the marginal effects of our estimated coefficients, our study identifies being female as having a 0.67% reduction in the probability of achieving at least good satisfaction compared to being male. We found that older age is associated with higher satisfaction, with individuals over 66 being 10.07% more likely to achieve at least good satisfaction compared to individuals in the reference age group (35 or under). Employed patients were 1.44% more likely to report high satisfaction compared to unemployed individuals. Patients admitted to a mental health institution were 4.14% less likely to have a high satisfaction compared to non-admitted patients, while patients able to work are 2.06% more likely to report high satisfaction levels compared to unable to work patients.

A unitary increase in the 1-to-6 scale for mental health self-assessment is associated with a 6.61% increase in the probability of reporting higher satisfaction. Longer contact length and longer time from last contact with services were both associated with negative satisfaction. Patients treated in the North region or in 2013 were respectively 2.75% and 5.42% more likely to report an at least good level of overall satisfaction compared to other patients.

Our study finds a number of novel results. First we find that service type affects patient satisfaction, with patients receiving talking therapies, and those who were prescribed medications being respectively 7.42% and 3.41% more likely to experience a higher satisfaction. Having a care plan was also associated with positive satisfaction, with patients having a care plan being 10.28% more likely to report higher satisfaction. Our model identifies that patients reporting an accommodation, a physical, or a financial need were respectively 4.51%, 1.94% and 3.08% less likely to achieve a high satisfaction level compared to other patients. The type of health professional most recently seen by the patient appeared to influence satisfaction, with being seen by a community psychiatric nurse, a social worker, or a mental health support worker leading to a 4.12%, 1.5% or a 2.93% increase in the probability of reporting an at least good assessment of overall satisfaction respectively. At a hospital level we found that a 1% increase in occupied beds was associated with a 7.2% decrease in the probability of reporting at least good overall satisfaction.

Our sensitivity analysis model adopting the alternative mapping for overall satisfaction in year 2013 identified comparable estimated coefficients, except for 2013 becoming negative, and with the percentage of occupied beds variable becoming non-significant (see model M3 in Table 4).

**Table 4:** Sensitivity analysis

Model	Patient and hospital characteristics model (M2)		Patient and hospital characteristics model with alternative satisfaction mapping (M3)	
N obs	28,288		28,288	
Variable	Coefficient	Standard error	Coefficient	Standard error
Year [2011]	− 0.02	0.02	− 0.03	0.02
Year [2012]	− 0.02	0.03	− 0.03	0.03
Year [2013]	0.23***	0.04	− 0.22***	0.04
North	0.11*	0.04	0.12*	0.05
South	− 0.02	0.05	− 0.01	0.05
Midlands and East	0.07	0.04	0.08	0.05
Patient-level characteristics				
Female	− 0.03*	0.01	− 0.03*	0.01
Mental health status	0.27***	0.01	0.27***	0.01
Admitted	− 0.17***	0.02	− 0.18***	0.02
Age 36–50	0.21***	0.02	0.2***	0.02
Age 51–65	0.27***	0.02	0.26***	0.02
Age over 66	0.42***	0.02	0.40***	0.02
Employed	0.06**	0.02	0.05**	0.02
Student	0.03	0.04	0.02	0.04
Voluntary	− 0.03	0.02	− 0.03	0.02
Length of contact with services	− 0.08***	0.01	− 0.08***	0.01
Last contact with services	− 0.18***	0.01	− 0.18***	0.01
Therapy: prescribed medications	0.14***	0.02	0.13***	0.02
Therapy: talking therapies	0.31***	0.01	0.30***	0.01
Having care plan	0.43***	0.02	0.42***	0.02
Being able to work	0.09***	0.02	0.09***	0.02
Physical health need	− 0.08***	0.01	− 0.08***	0.01
Accommodation need	− 0.19***	0.02	− 0.18***	0.02
Financial need	− 0.13***	0.01	− 0.13***	0.01
Community psychiatric nurse	0.17***	0.02	0.17***	0.02
Social worker	0.06*	0.03	0.07*	0.03
Psychiatrist	0.03	0.02	0.02	0.02
Mental health support worker	0.12***	0.02	0.12***	0.02
Occupational therapist	0.05	0.04	0.04	0.04
Hospital-level characteristics				
White	− 0.20	0.44	− 0.24	0.44
Mixed	0.4	0.81	− 0.30	0.82
Asian	− 0.71	0.57	− 0.77	0.58
Black	− 0.18	0.56	− 0.19	0.57
FTE staff	− 0.01	0.01	− 0.01	− 0.01
Percentage occupied beds	− 0.30	0.15*	− 0.31	0.15
MHRCI	0.05	0.03	0.05	0.03
Staff work-related stress	0.18	0.21	0.16	0.21
Foundation Trust status	− 0.01	0.02	− 0.01	0.02

Threshold	Estimate	Standard error	Estimate	Standard error
1|2	− 1.78	0.03	− 1.19	0.48
2|3	− 1.32	0.03	− 0.66	0.47
3|4	− 0.78	0.03	− 0.01	0.47
4|5	− 0.20	0.03	0.66	0.47
5|6	0.62	0.03	1.69	0.47
Diagnostics				
LogLik	− 40,071.62		− 41,004.76	
AIC	80,231.25		82,079.52	

Ordered probit model with alternative mapping. 2010 is the reference year, male is the reference gender, age < 35 is the reference age, “other ethnicity” is the reference ethnicity status. M2 is our reference model. M3 is used for sensitivity analysis

Significance is *p < .05, **p < .01, ***p < .001

Our alternative model specifications (not presented) identified the same significant variables as model M2, with the exception of percentage of occupied beds becoming not significant in the simplified probit model and in the linear model. In addition, in the linear model the dummies for the years 2011 and 2012 became significant, while female gender became non-significant. At a hospital level the linear model identified all ethnicity variables and MHRCI as positive and significant. Excluding the observations in the year 2013 led to no qualitative difference, except for female gender becoming non-significant. No significant changes were identified in the varying slopes model.

### Analysis of Variance and Provider Random Effects

**Fig. 1:**
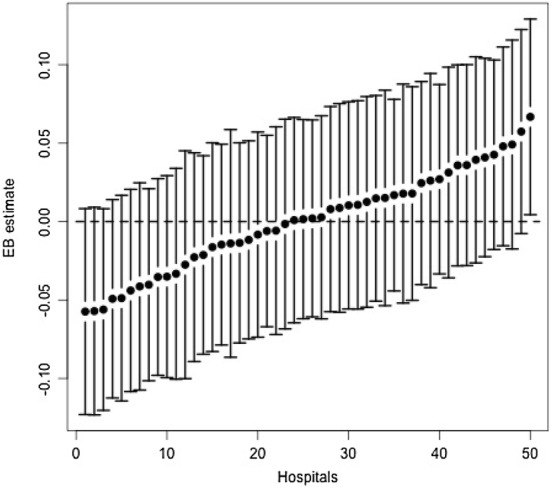
Empirical Bayes estimates with 95% confidence intervals of hospital-level residual variance for ordered probit model. The x-axis represents individual hospitals. The y-axis report Empirical Bayes estimates

**Fig. 3:**
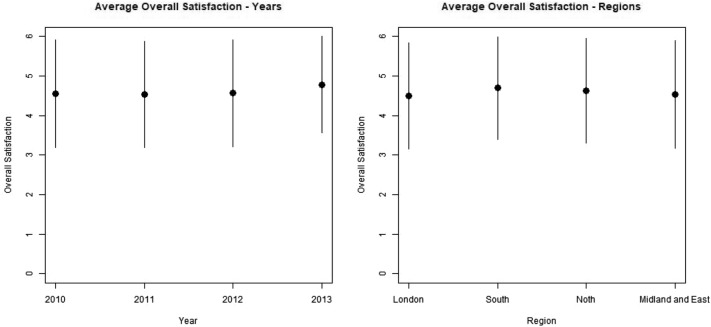
Hospital mean overall satisfaction across years (left) and across commissioning regions (right). Bold dots represent average values, lines represent standard deviation

**Fig. 4:**
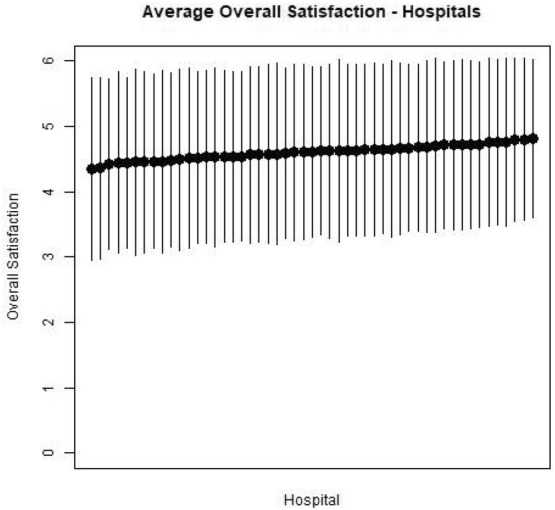
Mean overall satisfaction across hospitals. Bold dots represent average values, lines represent standard deviation. The x-axis represents individual hospitals. The y-axis report Empirical Bayes estimates

Figure 1 presents the Empirical Bayes for hospital-level residual variation estimated using model M2. Hospitals are ordered from left to right according to their performance on patient satisfaction after conditioning on covariates. Numbers on the x-axis indicate arbitrary numeric identifiers for individual hospitals. The y-axis indicates Empirical Bayes estimates. The whiskers of the graph represent the 95% confidence intervals of the estimated provider-specific effects. Hospitals with higher conditional variance in the estimated provider-effect will have wider confidence intervals compared to other providers. As shown in the figure, we identify the absence of overlaps in whiskers between the bottom 3 and top 1 performing hospital. This result highlights the presence of, albeit small, some statistically significant variation across providers, even once other covariates are accounted for.

## Conclusions

Our paper focused on identifying the factors associated with patient satisfaction with community mental health services in England via a multi-level analysis including both patient- and provider-level variables.

Our paper identified a number of novel results. First, we identify that treatment type affects satisfaction, with patients receiving talking therapies and prescribed medications reporting higher satisfaction. Second, we identify that a coordinated approach to care, as indicated by having a care plan, positively affects satisfaction. Third, our analysis highlights the need for integrated care, with patients reporting physical, financial or accommodation needs reporting lower satisfaction. Fourth, we identify that having a last interaction with a care professional closer to the community, such as a community psychiatric nurse, a social worker, or a mental health support worker, improves satisfaction. We interpret these results as evidence that a coordinated approach to care, higher care integration, and being treated closer to the community all lead to higher patient satisfaction.

By applying multi-level techniques to community mental health services, our study finds the presence of hospital-specific performance variation, even once other covariates are accounted for. We interpret these differences as resulting from different unobservable factors across hospitals such as variation in management styles and the organisation and design of community services.

The results presented in this analysis will be useful to policymakers in understanding what affects patient satisfaction in community mental health settings and in understanding how to use limited resources to effectively plan and co-ordinate care to meet patients’ expectations. In particular, our analysis identified the need to focus on the patient journey, providing a coordinated approach to care and ensuring the provision of integrated services.

Our work will be useful to hospital regulators in the monitoring and inspection of hospitals as variations in satisfaction might identify potential differences in quality of care. Particular attention should be given by regulators to understanding hospital-specific variation in patient satisfaction when planning regulatory activities.

Given the international interest towards providing mental health care in the community, our analysis might be useful for other countries aiming to identify what factors should be accounted for when planning the provision of care away from institutional settings.

Our study is subject to a number of limitations. Our dataset provides limited evidence on the role of ethnicity as these variables are not available at a patient-level. Our dataset is also affected by having a different scale of patient satisfaction for the year 2013. Lastly, being based on a repeated sample, our dataset does not provide pseudonomised patient identifiers and we are restricted to analysing repeated cross-sections.

Future research should consider how some of the harder to measure factors such as the quality and style of hospitals’ management impacts overall satisfaction. The importance of access to services, contact length and closeness to the community in affecting satisfaction suggests that additional attention should be given to understand the role that the patient journey has on overall satisfaction with services. Lastly, future research should focus on exploring the impact of different aspects of integrated care on patients’ satisfaction.

## Figures and Tables

**Fig. 2:**
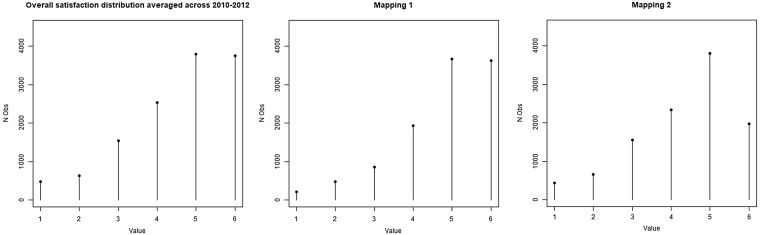
Histograms of overall satisfaction mapping. The plots represent the average overall satisfaction across the three available years (left), mapping 1 (centre), mapping 2 (right)

## Appendix

### Data Cleaning

The following responses to survey questions were reported as NA in our final dataset: “not applicable”, “not answered”, “don’t know/can’t remember”, “item not applicable”, “schedule not applicable” and “not answered”.

Answers to two option questions (“Yes” or “No”) were coded as 1 and 0 respectively. These questions included: (a) receiving prescribed medications, (b) receiving talking therapies, (c) having a care plan, (d) having a physical health need, (e) being able to work, (f) having an accommodation need, (g) having a financial need, (h) being admitted to a mental health hospital.

Answers referring to patient’s contact length with services were coded on a 0 to 3 scale, indicating “less than 1 year”, 1 indicating “1 to 5 years”, 2 indicating “6 to 10 years” and 3 indicating “more than 10 years”. Answers to patient’s last contact with services were coded on a 0 to 4 scale with 0 indicating “in the last month”, 1 indicating “1–3 months ago”, 2 indicating “4–6 months ago”, 3 indicating “7–12 months ago” and 4 indicating “more than 12 months ago”. Patients’ self-reported mental health was coded from 1 (“Very poor”) to 6 (“Excellent”).

The following variables were turned into (0, 1) dummy variables: (a) most recent health professional seen by the patient, (b) age group, (c) employment status.

### Overall Satisfaction Mapping

Overall satisfaction in year 2013 was mapped into a 6 point scale using two alternative mappings.

Mapping 1 was: ((10, 9) → 6, (8, 7) → 5, (6, 5) → 4, (4, 3) → 3, (2, 1) → 2, (0) → 1).

Mapping 2 was: ((10) → 6, (9, 8) → 5, (7, 6) → 4, (5, 4) → 3, (3, 2) → 2, (1, 0) → 1).

Figure 2 shows that mapping 1 underestimates the objective for low values of patient satisfaction, while the opposite holds true for mapping 2. Figure 3 shows overall satisfaction aggregated across years (left) and across regions (right). Figure 4 shows overall satisfaction averaged across hospitals, with lines representing standard deviation.

## Footnotes

1 The technical documentation of the Community Mental Health Survey does not explicitly indicate whether the sample is representative of the service user population. While the number of responses is large and the response rate is in line with other national health surveys, it is not possible to completely rule out the presence of non-response bias. See http://nhssurveys.org/wp-content/surveys/05-community-mental-health/03-instructions-guidance/2013/Survey%20guidance%20manual.pdf.

2 In reality NHS Mental Health Trusts (the legal entities) may comprise several different hospitals and may provide community services in many different localities, but for convenience we refer to these all as hospitals.

3 Mapping 1 was: ((10, 9) → 6, (8, 7) → 5, (6, 5) → 4, (4, 3) → 3, (2, 1) → 2, (0) → 1). Mapping 2 was: ((10) → 6, (9, 8) → 5, (7, 6) → 4, (5, 4) → 3, (3, 2) → 2, (1, 0) → 1).

4 Notice that in Fig. 4 the x-axis represents individual hospitals.
